# Regenerative potential of graphene oxide-chitosan nanocomposite combined with fetal bovine serum on healing of full-thickness skin wound in rats

**DOI:** 10.1186/s12917-025-04721-z

**Published:** 2025-05-07

**Authors:** Kamal H. Hussein, Mahmoud Soliman, Mahmoud S. Sabra, Hani Nasser Abdelhamid, Mahmoud Abd-Elkareem, Ahmed Abdelrahiem Sadek

**Affiliations:** 1 Department of Surgery, Anesthesiology and Radiology, Faculty of Veterinary Medicine, Assiut University, Assiut, 71516 Egypt; 2 Tissue Culture and Stem Cells Unit, Molecular Biology Researches & Studies Institute, Assiut University, Assiut, 71526 Egypt; 3 Department of Pathology and Clinical Pathology, Faculty of Veterinary Medicine, Assiut University, Assiut, 71516 Egypt; 4 Department of Pharmacology, Faculty of Veterinary Medicine, Assiut University, Assiut, 71516 Egypt; 5 Department of Chemistry, Faculty of Science, Imam Mohammad Ibn Saud Islamic University (IMSIU), Riyadh, 11623 Saudi Arabia; 6 Department of Cell and Tissues, Faculty of Veterinary Medicine, Assiut University, Assiut, 71526 Egypt

**Keywords:** Graphene oxide, Chitosan, Wound healing, Skin tissue engineering, FBS, Cytocompatibility

## Abstract

**Background:**

Delayed wound closure and non-healing wounds represent a problematic condition with health burden and an economic challenge. Therefore, different strategies have been developed, including skin tissue engineering, which aims to stimulate and support the wound healing process. In this study, the potential of graphene oxide (GO) and chitosan (CTS) biomaterial composite, with and without fetal bovine serum (FBS), was investigated to induce a full-thickness skin wound repair in rats.

**Methods:**

The GO-CTS composite was characterized using X-ray diffraction, transmission electron microscopy, and Fourier transforms infrared. Cytocompatibility was evaluated via an MTT assay with human endothelial cells (ECs) and mouse embryonic fibroblasts (MEFs) in vitro. The in vivo wound regeneration potential was assessed by creating an 8 mm full-thickness circular skin defect on the dorsal surface of the rat. The defects were randomly divided into control, GO-CTS, FBS, and GO-CTS/FBS groups, and were monitored grossly and histologically at days 7 and 21 after wound induction.

**Results:**

The GO-CTS material demonstrated high cytocompatibility, with cell viability recorded at 99.2% ± 5.7% for ECs and 110.5% ± 3.9% for MEFs. The highest proliferation rates were observed in the FBS (118.2% ± 2.1%) and GO-CTS/FBS (121.4% ± 4.4%) groups. In vivo, wound closure rates on day 21 were 85.5% ± 0.56% for GO-CTS, 87.5% ± 1.75% for FBS, and 91.5% ± 1.03% for GO-CTS/FBS, all significantly higher than the control group. Additionally, neovascularization, epithelialization, collagen deposition, and granulation tissue formation were more prominent in the treated groups, with skin appendages observed in the GO-CTS/FBS group.

**Conclusion:**

GO-CTS nanosheets with FBS represent a promising biomaterial for skin tissue engineering and can effectively initiate and support wound healing.

## Introduction

Injuries of the skin are a frequently reported affection of immense health care owing to the necessity of the skin in various physiological activities. The skin serves mainly as a physical barrier against external threats such as bacteria, fungi, viruses, radiation, and other harmful issues. Additionally, skin is crucial for vitamin D metabolism, regulation of body temperature, fluid balance, and immunological reactions [1, 2]. The occurrence of wounds resulting in damage to or removal of the skin, could predispose to scarring, wound infection, ulcer formation, and even gangrene [3, 4].

Skin has naturally evolved to heal wounds by restoring its protective barrier. Wound healing is a combinatorial process that needs the existence of suitable microenvironmental cues and a variety of interacting cells. The events of healing include orchestrated steps of inflammation, epithelialization, proliferation, angiogenesis, and remodeling. Throughout the healing process, fibroblasts, endothelial cells, platelets, and macrophages actively migrate, infiltrate, proliferate, and differentiate. This process leads to tissue growth and wound closure [4–7]. While natural skin regeneration often allows for normal healing, there are still several obstacles to rapid wound healing [1, 8]. These obstacles include infection, inadequate tissue oxygenation, limited vascularity, concurrent disease, and immunocompromised diseases [2, 9]. Hence, skin tissue engineering has emerged as a solution to hasten wound healing and overcome unfavorable healing outcomes. Skin tissue engineering implicates the integration of cells and growth factors into scaffolds to provide a favorable microenvironment for initiation of cell adhesion, migration, proliferation, and differentiation. The scaffold material should be biocompatible with suitable porosity [10, 11].

Two-dimensional nanomaterials such as graphene oxide (GO) are widely used in biomedicine, in particular tissue engineering [12, 13]. GO nanosheets are characterized by specific features such as high stability, good conductivity, high hydrophilicity, high drug loading capacity, and biocompatibility [14–17]. Thus, GO is incorporated alone or in combination with other materials in tissue engineering, including skin [2, 15, 18], bone [13], cartilage [19], skeletal muscles [20], and peripheral nerves [21]. It was also reported for photothermal treatment of wounds infected with pathogenic bacteria [22, 23].

Biopolymers have been considered a promising biomaterial for medical applications [6]. Chitosan (CTS) is a biopolymer derived from chitin and has desirable properties including excellent biodegradability, biocompatibility, antimicrobial activity, and minimal toxicity [24, 25]. CTS was fabricated into scaffolds that were applied in skin [26, 27], bone [28], cartilage [29], nerve [30], and cardiac [31] tissue repair.

Fetal bovine serum (FBS) is frequently utilized as a supplement for promoting growth in cell and tissue culture media. FBS is beneficial to most human and animal cell types due to its rich source of essential components needed for cell attachment, development, and proliferation [32, 33]. FBS is a sterile liquid obtained from clotted blood collected from pregnant cows and contains various components vital for the growth and survival of mammalian cell lines in vitro. Despite the availability of standardized serum-free media, FBS remains the most widely used additive in cell culture media [34]. FBS effectively stimulated fibroblast migration and proliferation in vitro, suggesting its promising use in wound repair applications [35]. The present work investigated the efficacy of graphene oxide/chitosan (GO-CTS) nanosheets with and without FBS for regeneration of skin wounds in rats.

## Materials and methods

This study includes the fabrication and characterization of the nanocomposite, in vitro cytotoxicity testing, and in vivo testing of the developed material in a rat model. Rats were received from the House of Experimental Animals, Veterinary Teaching Hospital, Faculty of Veterinary Medicine, Assiut University, Assiut, Egypt. The Assiut Veterinary Medicine Research Ethics Committee, Assiut University, Assiut, Egypt, approved the various steps of the experimental work (No. 06/2024/0264) in compliance with ARRIVE regulations and OIE standards for the care and use of animals in research and education.

### Chemicals

Natural graphite powder (20 + 84 mesh, 99.9%) was supplied by Alfa Aeser (Great Britain). Potassium permanganate (KMnO_4_), chitosan (CTS, 75% deacetylated), and sodium nitrate were supplied by Sigma Aldrich (Germany). Sulfuric acid (60%) was purchased for Al-Naser Co. (Egypt).

### Synthesis of GO-CTS nanocomposite

Graphene oxide was synthesized via Hummer’s method [36] based on the strong oxidation of natural graphite, as detailed in previous work by Ibrahim, Abdelhamid [37]. Briefly, 1 g of natural graphite was added into a flask surrounded with an ice bath containing sodium chloride and exposed to magnetic stirring. Subsequently, 10 mL of nitric acid (69–72%) containing 0.5 g sodium nitrate was added to the flask. Sulfuric acid (15 mL, 96.0%) and potassium permanganate (3 g, 99%) were incrementally introduced into the mixture. The temperature was maintained below 0 °C via an ice-sodium chloride bath. Following 2 h of magnetic stirring, 200 mL of distilled water was gradually added to the mixture, resulting in a dark brown colloidal suspension. Hydrogen peroxide (30–32%, 15 mL) was added to eliminate the surplus permanganate until effervescence ceased. Following additional stirring for 30 min, the mixture was filtered and repeatedly rinsed with a 5 wt% HCl solution to eliminate sulfate ions. GO-CTS material was prepared following Abdelhamid and Wu [38] with modification. Chitosan was dissolved at 1 wt% in water containing 1 wt% acetic acid to prepare the solution. GO (1 wt%) was then added and the solution was stirred overnight till obtaining a homogenous colloidal solution of GO-CTS.

### Characterization instruments

X-ray diffraction (XRD) analysis was conducted using a Bruker AXS D8 Advance System (Germany). The powdered sample was placed on a zero-background holder. Transmission electron microscopic (TEM) imaging for GO was performed on a JEM-2100 TEM (JEOL, Japan). The image was obtained from a diluted dispersion in an aqueous solution. A 10 μL aliquot of the dispersion was deposited onto a copper grid coated with a carbon film. Fourier transform infrared (FT-IR) spectra were collected on attenuated total reflectance FT-IR (ATR-FTIR) spectra (UK, Varian 610-IR). The sample was analyzed via direct deposition on a diamond piece of the Golden Gate ATR accessory.

### Cytotoxicity assay

For mitigating the detrimental effects of leached chemicals, extracts were generated from the GO-CTS nanocomposite. Ethylene oxide gas was used to sterilize the nanomaterials, which were subsequently placed in serum-free Dulbecco’s Modified Eagle’s Medium (DMEM; Invitrogen, USA) containing 1% penicillin/streptomycin (p/s; Gibco, USA) at 37 °C with shaking at 120 rpm for 72 h. The incubation was conducted with a culture medium at a concentration of 0.2 g/mL [39]. After incubation, the supernatant was collected, centrifuged to obtain conditioned extracts, filtered through 0.4 μm filters, and preserved at 4 °C for subsequent cytotoxicity analysis.

Human endothelial cells (ECs), specifically the EA.hy926 cell line and mouse embryonic fibroblasts (MEFs), were cultured in DMEM containing 10% fetal bovine serum (Hyclone, USA) and 1% p/s under standard conditions (37 °C, 5% CO_2_) in a humidified incubator. Cells were harvested at 70% confluency by trypsinization for further experimental procedures. For the assay, cells were cultured at 1.5 × 10^4^ cells per well in a 48-well plate and incubated for 24 h in complete culture medium (DMEM supplemented with 10% FBS and 1% p/s) to promote adhesion. After aspiration of the medium, 500 μL of either the conditioned or control medium was placed into each well.

For the control groups, the negative control wells contained cells grown in DMEM containing 1% p/s (without FBS), whereas the positive control group (toxic group) was treated with 20% dimethyl sulfoxide (DMSO) to induce extensive cell lysis. Additionally, a 10% FBS-treated group was included to assess the effect of serum on cell viability. The extracts’ effect on cellular response was assessed after 24 h of incubation using the (3-[4,5-dimethylthiazol-2-yl]-2,5 diphenyl tetrazolium bromide) (MTT) assay. Metabolically active cells convert MTT dye into purple formazan crystals. In brief, 50 μL of MTT solution (5 mg/mL; Sigma-Aldrich, USA) was introduced to each well, followed by 4 h of incubation at 37 °C. Following incubation, the medium with the MTT dye was discarded, and 250 μL of DMSO was added. After incubating for 10 min, 100 μL aliquots were transferred to a 96-well plate, and absorbance was recorded at 570 nm using a spectrophotometer. Cell viability was calculated relative to negative control.

### Skin wound healing model induction

In this study, female rats (*n* = 20, 8 weeks, 200–250 g) were utilized to assess the efficacy of GO-CTS nanosheets alone or combined with FBS in enhancing the skin healing cascade. Rats were housed individually in standard conditions and provided with a diet of commercial rat chow and *ad libitum* access to water. A 7-day acclimatization period was provided to the animals prior to surgery. The model of skin healing was conducted as described by Soliman, Sadek [2] and Hussein, Abdelhamid [15]. A total of 40 skin defects were placed in a random manner into four groups; the control group, the GO-CTS treated group, the FBS treated group, and the GO-CTS/FBS treated group. Animals were subjected to isoflurane inhalation anesthesia (Forane: AbbVie, England) through the usage of an induction chamber at a dose of 2–3.5% in 100% oxygen for induction and a nose cone at a dose of 1.5–3.5% for maintenance anesthesia. Aseptic preparation of the skin on the dorsum was done, and the animals were placed in sternal recumbency. An 8 mm in diameter circular full-thickness model of skin wounds had been performed bilaterally on the dorsum, with a distance of 2 cm between the wounds. The skin defects were treated with 0.3 mL of saline (control defects), GO-CTS, FBS (0.3 mL), or GO-CTS/FBS. Rats then returned to their boxes and were allowed to move without limitations with careful monitoring for any complications throughout the study. The animals were euthanized on days 7 and 21 after induction of skin wounds via the use of isoflurane followed by decapitation.

### Gross evaluation of the wound

The wounds were digitally photographed on days 0, 7, and 21, with a calibrated ruler placed beside the wounds to enable accurate digital measurement of the photos. Wound closure was measured using ImageJ software and presented as a percentage, following the method previously outlined [2], using the formula below:

$$\begin{array}{l}{\rm{Wound}}\,{\rm{closure}}\,\left( {\rm{\% }} \right)\,{\rm{ = }}\\\frac{{{\rm{Initial}}\:{\rm{wound}}\:{\rm{area}} - {\rm{Wound}}\:{\rm{area}}\:{\rm{at}}\:{\rm{each}}\:{\rm{time}}\:{\rm{point}}}}{{{\rm{Initial}}\:{\rm{wound}}\:{\rm{area}}}}\: \times \:100\end{array}$$

### Histological examination

The skin tissue samples were obtained then fixed in 10% neutral buffered formalin. After fixation, the samples underwent routine processing, were embedded in paraffin, sectioned, and stained using hematoxylin and eosin. The epithelial gap between the left and right wound edges was measured in micrometers using ImageJ, with five wounds being analyzed per group. The number of inflammatory cells, and the number and average diameter of the new blood vessels were also counted. All histological analyses were performed on five images per wound sample to obtain the statistical results [2, 40].

### Histochemical staining of collagen

Gomori’s trichrome stain was applied to investigate the wound healing process, highlighting the extent of collagen deposition. The tissue sections embedded in paraffin were first deparaffinized using xylene, then gradually rehydrated through a sequence of ethanol solutions, followed by immersion in phosphate-buffered saline, and finally rinsed with distilled water. The sections were then stained with Gomori’s trichrome stain following the manufacturer’s guidelines, dehydrated through a series of alcohol solutions, cleared in xylene, and mounted. Collagen staining with green color was then verified under the microscope. Measurement of granulation tissue thickness was performed on a scale of 1–3: 1 = mild; 2 = moderate; 3 = complete [40]. The percentage of collagen-positive areas was assessed in ImageJ using a threshold area fraction method. Collagen content was quantified as a percentage of the total pixel count in the field of view and reported as the mean ± standard deviation [41].

### Statistical analysis

One-way ANOVA followed by Tukey’s post hoc test was conducted for statistical analysis in IBM SPSS software (version 21, USA), and results with a *p*-value less than 0.05 were considered statistically significant.

## Results

### GO-CTS nanocomposite synthesis and characterization

**Fig. 1:**
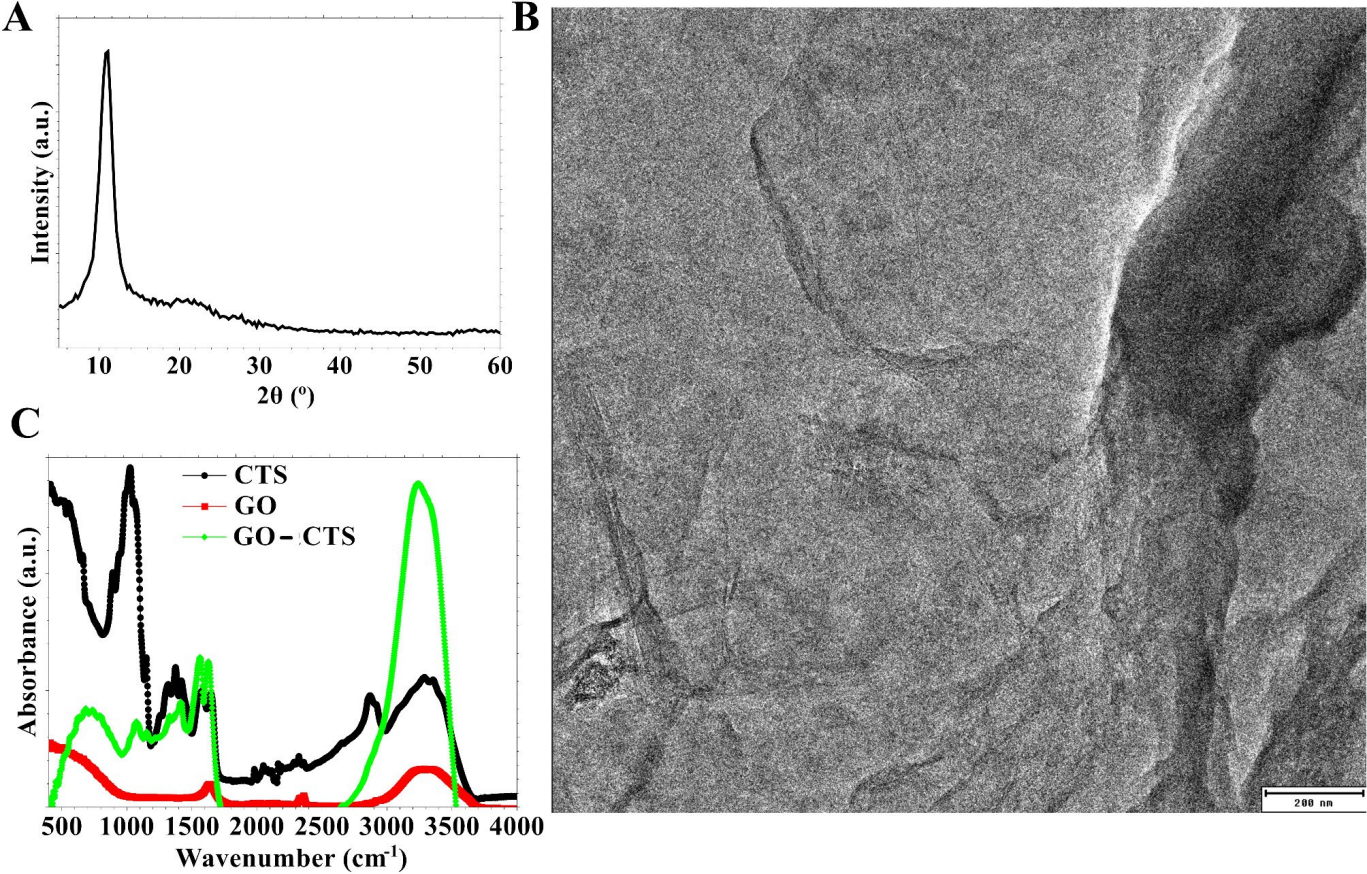
Characterization of GO-CTS biomaterials using (**A**) XRD, (**B**) TEM image, and (**C**) FT-IR

GO was synthesized via strong oxidation of graphite following Hummer’s method. It was then dispersed into a solution of CTS producing GO-CTS biomaterial. The material was analyzed using XRD (Fig. 1A), TEM image (Fig. 1B), and FT-IR spectra (Fig. 1C).

The phase and material’s crystallinity were characterized using XRD (Fig. 1A). The XRD spectrum for GO displays a prominent Bragg diffraction peak at 10.9°, matching the Miller index of the (002) plane. The observed diffraction peaks refer to d-spacing of 0.9 nm. The shape of GO was evaluated using a TEM image (Fig. 1B). The transparency observed in the TEM image of GO suggests that it consists of a few 2D layers. Additionally, the TEM image reveals a crumbling appearance of GO.

The functional groups and interactions inside GO-CTS were determined using FT-IR (Fig. 1C). GO displays vibrational bands at 3280 cm^-1^, and 1640 cm^-1^ which correspond to O − H stretching, and C = O stretching of carboxylic acid groups. The observed FT-IR peak at 2350 cm^-1^ refers to CO_2_ adsorbed into the materials. The FT-IR spectra of CTS exhibit distinctive bands at 3300 cm^-1^, 2870 cm^-1^, 1650, 1560 cm^-1^, 1380 cm^-1^, and 1020 cm^-1^ O − H stretching, C − H/ *N* − H stretching, C = O stretching, *N* − H bending, CH_2_ − OH, and C − O, respectively. GO-CTS composite displays the distinctive FT-IR characteristics peaks for both materials, i.e. GO and CTS. Nevertheless, the vibrational bands of C = O and C − O for CTS show shift in their wavenumber due to the interactions with GO via hydrogen bonds.

### Cytotoxicity assay

**Fig. 2:**
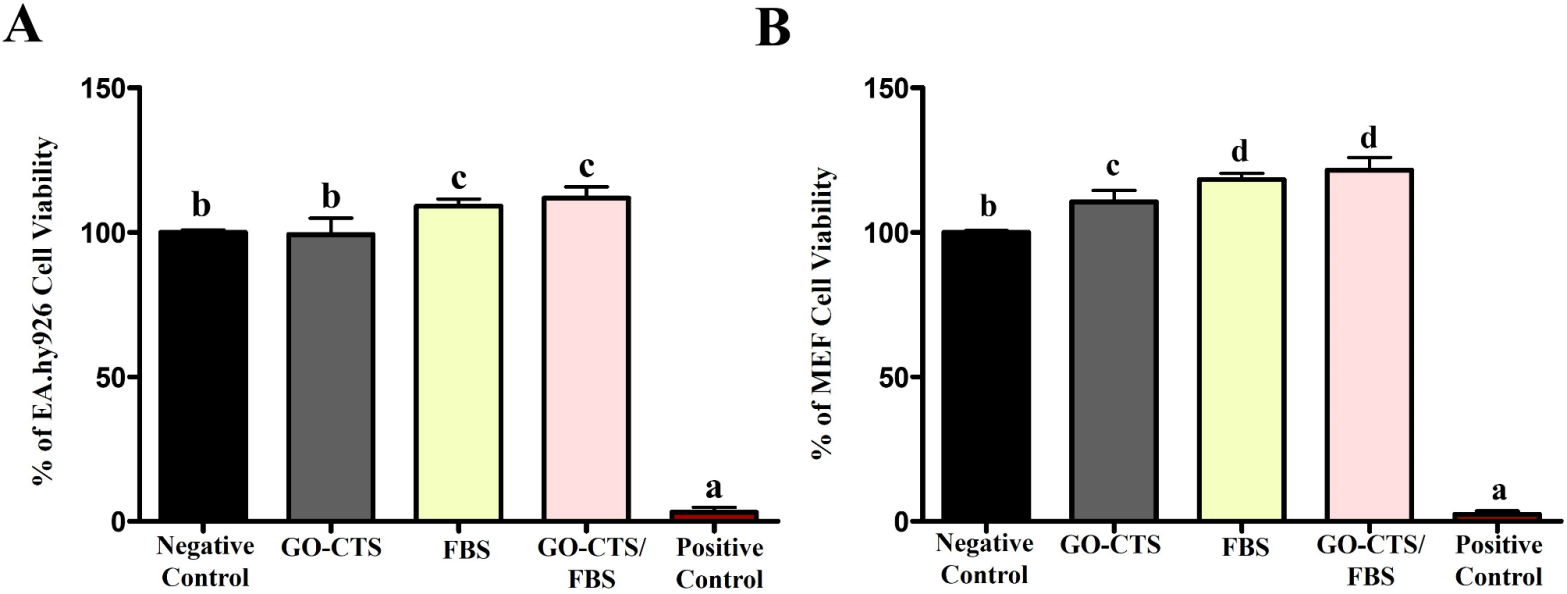
Cytotoxicity assay in vitro. MTT assay for cell viability of EA.hy926 endothelial cells (**A**) and MEF (**B**) cultured using extracts of GO-CTS, FBS, or GO-CTS/FBS for 24 h, compared to the negative control. Error bars represent the means ± standard deviation (*n* = 8 for each time point), with differences assessed using one-way ANOVA followed by Tukey’s HSD post hoc test. Groups marked with different letters represent statistically significant differences (*p* < 0.05), whereas groups sharing the same letter show no significant difference. The assay was done in triplicate

MTT assay indicated no significant difference in viability between EA.hy926 endothelial cells grown in complete DMEM and those treated with conditioned extracts from the GO-CTS material after 24 h (Fig. 2A). Furthermore, preconditioned media containing GO-CTS and supplemented with FBS demonstrated a higher percentage of cell proliferation compared to other groups. The positive control group (treated with DMSO) showed a significant reduction in viability at 3.1% ± 1.8%. Cells exposed to the GO-CTS-containing extracts exhibited a viability of 99.2% ± 5.7%. Notably, cells treated with FBS alone showed a viability of 109% ± 2.5%, and those exposed to the combination of GO-CTS and FBS demonstrated the highest viability at 111.8% ± 3.9% with no significant difference with the FBS alone.

In the analysis of MEF, the results paralleled the previous findings (Fig. 2B). The negative control group (DMEM) maintained a baseline cell viability set at 100%. The positive control group, treated with DMSO, displayed a significant reduction in cell viability, recorded as 2.4% ± 1.2%. Cells exposed to GO-CTS demonstrated a viability of 110.5% ± 3.9%, while cells treated with FBS alone reached 118.2% ± 2.1%. Importantly, the combination of GO-CTS and FBS yielded the highest cell viability, recorded at 121.4% ± 4.4%, closely aligned with the endothelial cell findings.

### Assessment of the wound closure in vivo

**Fig. 3:**
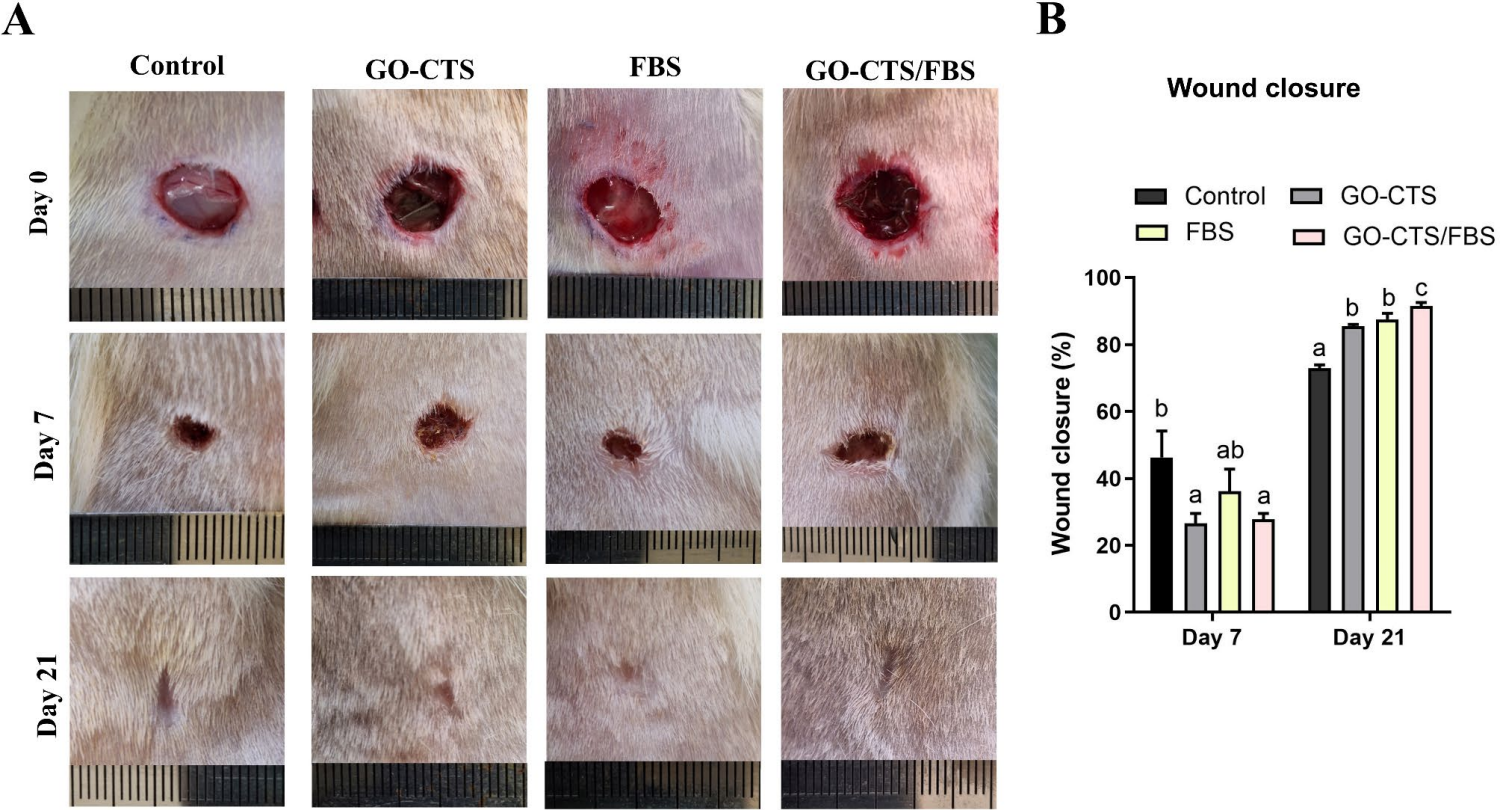
Gross assessment of skin wounds treated with or without GO-CTS, FBS, or GO-CTS/FBS. (**A**) Images showing skin wounds from the control and treated groups at days 0, 7, and 21. (**B**) Wound closure was quantified as a percentage through ImageJ analysis. Groups labeled with different letters represent statistically significant differences (*p* < 0.05), whereas groups sharing the same letter show no significant difference. Differences were evaluated using one*-*way ANOVA followed by Tukey’s HSD post hoc test

Wounds treated with GO-CTS, FBS, or GO-CTS/FBS exhibited a significantly accelerated healing process compared to the control group. The wound closure was not visibly observed on day 7. However, complete wound closure was observed in GO-CTS, FBS, or GO-CTS/FBS treated groups on day 21 and closure rates were recorded at 85.5% ± 0.56%, 87.5% ± 1.75%, or 91.5% ± 1.03%, respectively (Figs. 3A and B). Among the treatments, GO-CTS/FBS combination demonstrated the most pronounced effect, leading to the fastest closure rate, indicating a synergistic effect of the combined treatment. (Fig. 3B). A notable increase in hair growth was observed in the wound area following treatment with GO-CTS/FBS (Fig. 3A). These findings highlight the effectiveness of GO-CTS, FBS, and particularly their combination in enhancing wound healing and tissue regeneration.

### Histological findings on re-epithelization, inflammation and angiogenesis

To monitor the healing process, histological evaluations of the wounds were carried out on days 7 and 21. This involves assessing various aspects including re-epithelialization, inflammation, and angiogenesis.

**Fig. 4:**
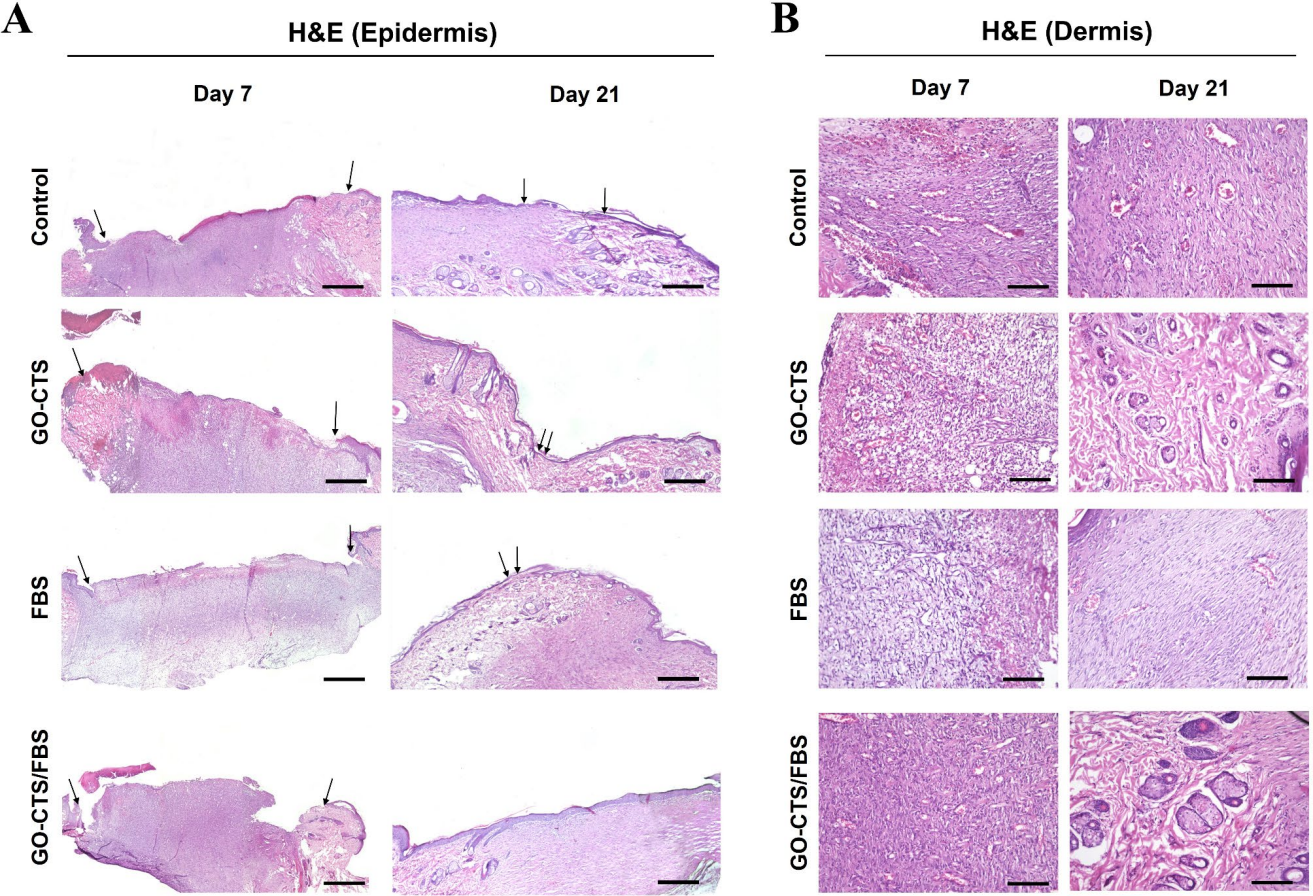
Histological evaluation of skin wounds in rats treated with or without GO-CTS, FBS, or GO-CTS/FBS. (**A**) and (**B**) Wound sections from control or GO-CTS, FBS, or GO-CTS/FBS -treated wounds collected on days 7 and 21 and stained by Hematoxylin and Eosin. Arrows highlight the epithelial layer. In (**A**), the scale bar measures 100 μm, and in (**B**), it measures 50 μm

Re-epithelialization was assessed by measuring the epithelial gap between the growing epidermis at the wound edges. By day 7, no significant difference was observed between the control and treated groups. (Figure 4A A). However, it was significantly enhanced following treatment with GO-CTS, FBS, or GO-CTS/FBS, as evidenced by a reduction in the epithelial gap by day 21 (Fig. 4A A). Notably, the GO-CTS/FBS-treated wounds exhibited complete re-epithelialization, forming a well-structured epithelial layer with evidence of skin appendage regeneration (such as the formation of hair follicles and sebaceous glands). In contrast, the epithelial layers in the GO-CTS and FBS groups appeared thin and incomplete, lacking the fully developed four-strata architecture. The control group displayed incomplete wound healing, with a persistent epithelial gap and delayed tissue regeneration (Fig. 4A).

**Fig. 5:**
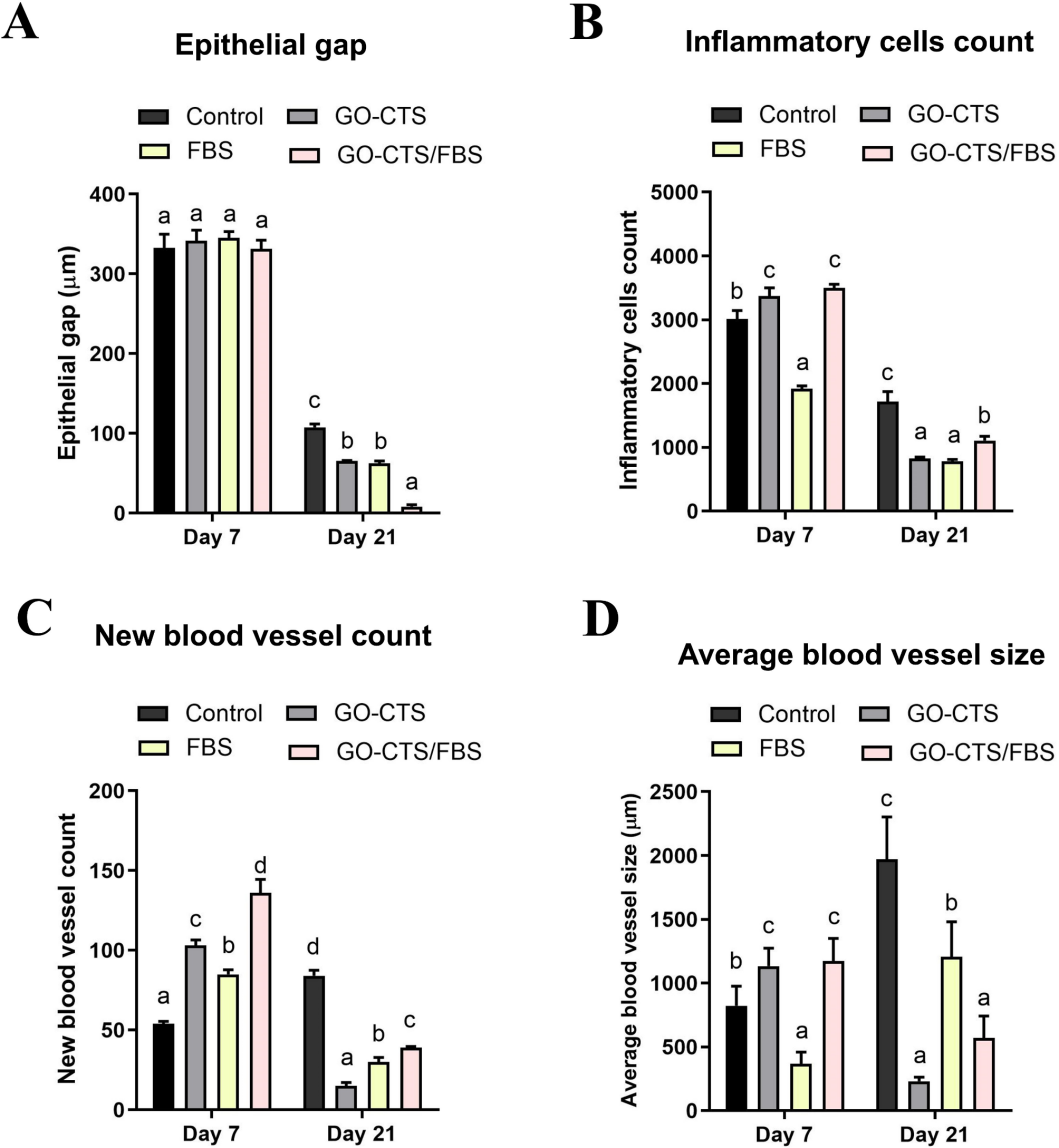
Histomorphometric analysis of the newly formed tissue in vivo. (**A**) The epithelial gap between the two wound edges as well as (**B**) the count of inflammatory cells at the wound site were quantified using ImageJ. (**C**) The number of new blood vessels was quantified in 5 images per section. (**D**) The size of the new blood vessels was assessed with ImageJ. Groups marked with different letters represent statistically significant differences (*p* < 0.05), whereas groups sharing the same letter show no significant difference. Differences were evaluated using one*-*way ANOVA followed by Tukey’s HSD post hoc test

Analysis of inflammatory cell infiltration revealed an initial influx of inflammatory cells into the wound area in the GO-CTS and GO-CTS/FBS groups on day 7, while the FBS group showed no significant inflammatory response (Figs. 4B and 5B). By day 21, the number of inflammatory cells had decreased significantly in all treated groups, indicating resolution of inflammation and progression of tissue remodeling (Figs. 4B and 5B). However, the control group showed a persistent presence of inflammatory cells, indicating delayed healing (Fig. 5B).

Angiogenesis was significantly enhanced in the treated groups by day 7 as the number of the newly formed blood vessels was markedly increased in the GO-CTS, FBS, and GO-CTS/FBS groups compared to the control group (Fig. 5C). Additionally, blood vessel size was significantly increased in the GO-CTS and GO-CTS/FBS groups, but not in the FBS-treated wounds (Fig. 5D).

However, by day 21, the number and size of newly formed blood vessels was significantly decreased in the treated wounds (Fig. 5C and D), whereas the control group showed a notable increase in blood vessel number and size (Fig. 5C and D). These findings suggest that GO-CTS and FBS promote the early proliferative phase of wound healing by stimulating angiogenesis.

### Histological evaluation of the granulation tissue

**Fig. 6:**
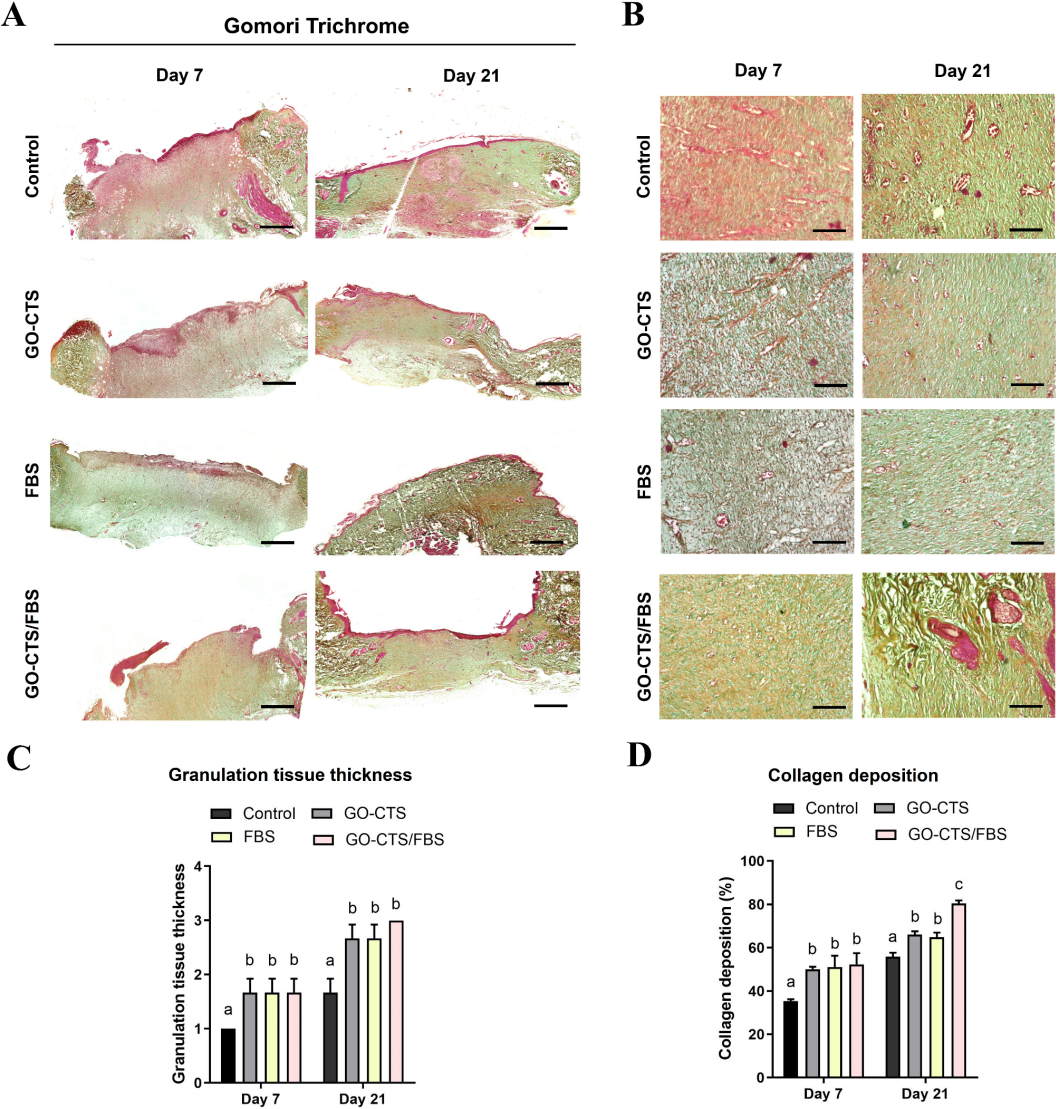
Histochemical evaluation of skin wounds in rats treated with or without GO-CTS, FBS, or GO-CTS/FBS. (**A**) and (**B**) Gomori’s Trichrome stained skin sections from control and GO cellulose-treated wounds obtained on days 7 and 21. In image (**A**), the scale bar is 100 μm, and in image (**B**), it is 50 μm. (**C**) Granulation tissue thickness was assessed using a scale of 1–3, as mentioned in the Materials and Methods. (**D**) Collagen deposition was quantified using ImageJ. Bars sharing the same letter indicate values that are not significantly different. Differences were evaluated using one*-*way ANOVA followed by Tukey’s HSD post hoc test

Gomori’s trichrome staining was applied to the wound sections to assess and quantify collagen deposition and granulation tissue formation at the wound center. On day 7 post-wounding, granulation tissue thickness showed no significant differences among the GO-CTS, FBS, GO-CTS/FBS groups, and the control group. At day 21 post wounding, the GO-CTS, FBS, and GO-CTS/FBS groups displayed notably thick and well-formed granulation tissue (Fig. 6A-C) and higher amounts of collagen content with a dense arrangement and staining of collagen fibers, in comparison to the control group (Fig. 6D).

## Discussion

Skin wounds with an impaired healing process are a significant health issue with considerable life and economic consequences. In recent decades, skin tissue engineering has aimed to overcome complications of non-healing wounds. Consequently, various scaffold materials have been explored to support and induce regeneration [2, 10, 11, 42]. Among these, GO and CTS have gained attention in tissue engineering due to their unique properties, including biocompatibility and the ability to promote cell migration, adhesion, and proliferation [2, 42–44]. When GO is combined with other compounds such as cellulose, it forms a composite material with the potential to enhance wound healing [2, 15, 18]. Additionally, CTS integrated into nanocomposites with materials such as fucoidan, pullulan, and gallium, has been shown to stimulate the process of wound healing [42, 44].

The synthesis and characterization of GO and its composite with CTS yield significant insights into the structural and functional attributes of the GO-CTS biomaterial. The findings from XRD (Fig. 1A), TEM (Fig. 1B), and FT-IR (Fig. 1C) investigations collectively validate the effective synthesis of GO, its incorporation with CTS, and the creation of a GO-CTS composite.

The XRD investigation exhibited a significant Bragg diffraction peak at 10.9°, corresponding to the (002) plane of GO, aligning with prior results that confirm the effective oxidation of graphite (Fig. 1A). The determined d-spacing of 0.9 nm signifies the incorporation of oxygen-containing functional groups during the oxidation process, a characteristic feature of GO synthesis. The interlayer spacing relative to pure graphite (about ∼ 0.34 nm) is ascribed to the presence of these functional groups, which concurrently improve the material’s hydrophilicity and dispersibility in aqueous conditions [14].

The TEM image further substantiates the development of GO, exhibiting a few-layered two-dimensional structure characterized by a translucent and crumpled appearance (Fig. 1B). This morphology is typical of GO sheets. The deformed structure indicates the existence of flaws and functional groups, which are crucial for interactions with other materials, such as CTS, in composite synthesis.

The FT-IR spectra offer essential insights into the functional groups present in GO, CTS, and the GO-CTS composite (Fig. 1C). The vibrational bands detected for GO, including O − H stretching (3280 cm⁻¹) and C = O stretching (1640 cm⁻¹), validate the existence of hydroxyl and carboxyl groups, which are incorporated during the oxidation process. These functional groups enhance the material’s hydrophilicity and promote its contact with CTS via hydrogen bonding and electrostatic interactions [16]. The FT-IR spectra of CTS display distinctive peaks associated with O − H stretching, C − H/*N* − H stretching, and C = O stretching, among others, aligning with its polysaccharide structure. The FT-IR analysis of the GO-CTS composite confirms the successful conjugation of GO and CTS, as indicated by the existence of distinctive peaks from both substances. The alterations in the vibrational bands of C = O and C − O in CTS indicate the establishment of hydrogen bonds between GO and CTS.

The results of XRD, FT-IR, and TEM image validate the effective synthesis of GO and its incorporation with CTS to create a GO-CTS composite. The identified structural and functional characteristics, including the enhanced d-spacing, crumpled morphology, and hydrogen bonding interactions, underscore the potential of this biomaterial for diverse biomedical applications, such as wound healing.

Furthermore, FBS is widely utilized as a supplement in cell culture media due to its abundance of growth factors, hormones, and nutrients crucial for promoting cell growth and proliferation [32, 45, 46]. While FBS is primarily used in vitro, there is limited evidence of its potential role in accelerating wound healing in vivo. Therefore, this study investigated the effects of GO-CTS and FBS on wound healing. We demonstrated that GO-CTS with FBS is a promising composite that significantly improves wound healing in a full-thickness skin wound model in vivo.

Cytocompatibility is crucial for skin tissue engineering to ensure that biomaterials support in vivo fibroblast and endothelial cell growth and adhesion [15]. Here, the cytotoxicity of the designed materials was tested on human ECs and MEF cells. The results indicated that the materials are cytocompatible, which is consistent with previous studies [2, 15, 47–49].

The ability of these biomaterials to promote regeneration depends on the host’s cellular reaction to the implanted materials, which triggers the influx of various immune cells [50]. The early recruitment of inflammatory cells, notably neutrophils and macrophages, plays a key role in wound cleaning and debris removal. Subsequently, during the proliferative and remodeling phases, macrophages promote angiogenesis, fibroblast proliferation, and collagen matrix deposition. As healing progresses, the number of inflammatory cells decreases due to an increase in fibroblasts [2, 51]. Aligned with this, our findings on day 7 displayed inflammatory cell infiltration in the wounds treated with GO-CTS and GO-CTS/FBS.

Neovascularization is a vital aspect in wound healing, as it supplies essential nutrients and oxygen to the healing tissue while removing waste products and promoting granulation tissue formation. It arises from pre-existing blood vessels through mechanisms driven by growth factors, inflammatory mediators, and extracellular matrix remodeling [52]. Our study suggests that GO-CTS and FBS enhance wound healing by promoting blood vessel formation, as reflected by a greater number of newly formed blood vessels in the early proliferation phase and larger vessel diameters in the later stages of the healing process.

Collagen, primarily synthesized by fibroblasts, serves as the key extracellular matrix component necessary for granulation tissue formation and the remodeling phase of wound healing [4, 53, 54]. In our study, collagen formation was minimal during the early phase of healing process, as the wounds were transitioning from inflammation to proliferation. As healing progressed, collagen became more mature and densely packed, and normal skin structures began to reappear. These observations suggest that treatment with GO-CTS and FBS may promote more efficient wound healing by enhancing granulation tissue formation and organization and collagen deposition. Moreover, the synergistic effect of GO-CTS and FBS ultimately leads to better tissue repair and regeneration compared to GO-CTS or FBS alone.

The favorable microenvironment created by GO-CTS and FBS in this study likely expedited wound closure and minimized the epithelial gap between wound edges. Inflammatory cell infiltration in the wound area stimulates the release of chemotactic and growth factors, initiating the proliferation of cells necessary for healing [50, 51, 53]. GO and CTS demonstrated immunomodulatory effects, promoting the migration of inflammatory cells that produce interleukin-1 (IL-1), IL-6, IL-8, platelet-derived growth factor (PDGF), transforming growth factor-β (TGF-β), and vascular endothelial growth factor (VEGF). These factors are critical in promoting healing cascades, including neovascularization, fibroblast proliferation, and collagen deposition [2, 12, 50, 55, 56]. Additionally, the surface properties and chemical composition of the GO-CTS material may enhance cell adhesion, proliferation, and migration [12, 15, 57]. FBS contains a variety of cytokines and growth factors that stimulate cell proliferation, migration, and differentiation, contributing to the tissue repair. These key factors include epidermal growth factor, fibroblast growth factor, insulin-like growth factor, PDGF, TGF-β, and IL- 1, − 2, − 4, -6, -8, and − 10 [50, 58]. All these factors promote the migration and proliferation of epithelial cells from the wound edges across the remaining basement membrane of the skin wound [53, 54].

## Conclusion

GO-CTS is a biocompatible nanocomposite capable of supporting the in vitro growth of fibroblasts and endothelial cells. Moreover, the vivo application of GO-CTS, with or without the addition of FBS, significantly enhances the healing process of full-thickness skin wounds in a rat model. These findings could contribute to the development of a fast and effective wound healing agent for clinical use.

However, this experimental study has certain limitations, including the lack of assessment of material biodegradation, a small in vivo sample size, and insufficient investigation of the possible cellular and molecular mechanisms underlying the material’s stimulatory effects. Therefore, future research should focus on evaluating the biodegradation of the GO-CTS nanocomposite as a scaffold for skin tissue regeneration. Additionally, the in vivo fate of its degradation byproducts must be assessed. Further studies should also be conducted on a larger sample size and animal models, including different skin wound types such as burn injuries and wounds in diabetic animals.

## Data Availability

No datasets were generated or analysed during the current study.

## Abbreviations

CTS: Chitosan
DMSO: Dimethyl sulfoxide
DMEM: Dulbecco’s Modified Eagle’s Medium
MEFs: Embryonic fibroblasts
FBS: Fetal bovine serum
GO: Graphene oxide
GO-CTS: Graphene oxide chitosan
ECs: Human endothelial cells
IL: Interleukin
PDGF: Platelet derived growth factor
TGF-β: Transforming growth factor-β
